# Nutrient acquisition by symbiotic fungi governs Palaeozoic climate transition

**DOI:** 10.1098/rstb.2016.0503

**Published:** 2017-12-18

**Authors:** Benjamin J. W. Mills, Sarah A. Batterman, Katie J. Field

**Affiliations:** 1 School of Earth and Environment, University of Leeds, Leeds LS2 9JT, UK; 2 School of Geography, University of Leeds, Leeds LS2 9JT, UK; 3 Priestley International Centre for Climate, University of Leeds, Leeds LS2 9JT, UK; 4 Centre for Plant Sciences, School of Biology, University of Leeds, Leeds LS2 9JT, UK; 5 Smithsonian Tropical Research Institute, Ancon, Panama

**Keywords:** carbon dioxide, oxygen, evolution, mycorrhizal symbiosis, climate, Palaeozoic

## Abstract

Fossil evidence from the Rhynie chert indicates that early land plants, which evolved in a high-CO_2_ atmosphere during the Palaeozoic Era, hosted diverse fungal symbionts. It is hypothesized that the rise of early non-vascular land plants, and the later evolution of roots and vasculature, drove the long-term shift towards a high-oxygen, low CO_2_ climate that eventually permitted the evolution of mammals and, ultimately, humans. However, very little is known about the productivity of the early terrestrial biosphere, which depended on the acquisition of the limiting nutrient phosphorus via fungal symbiosis. Recent laboratory experiments have shown that plant–fungal symbiotic function is specific to fungal identity, with carbon-for-phosphorus exchange being either enhanced or suppressed under superambient CO_2_. By incorporating these experimental findings into a biogeochemical model, we show that the differences in these symbiotic nutrient acquisition strategies could greatly alter the plant-driven changes to climate, allowing drawdown of CO_2_ to glacial levels, and altering the nature of the rise of oxygen. We conclude that an accurate depiction of plant–fungal symbiotic systems, informed by high-CO_2_ experiments, is key to resolving the question of how the first terrestrial ecosystems altered our planet.

This article is part of a discussion meeting issue ‘The Rhynie cherts: our earliest terrestrial ecosystem revisited’.

## 1. Introduction

**Figure 1.**
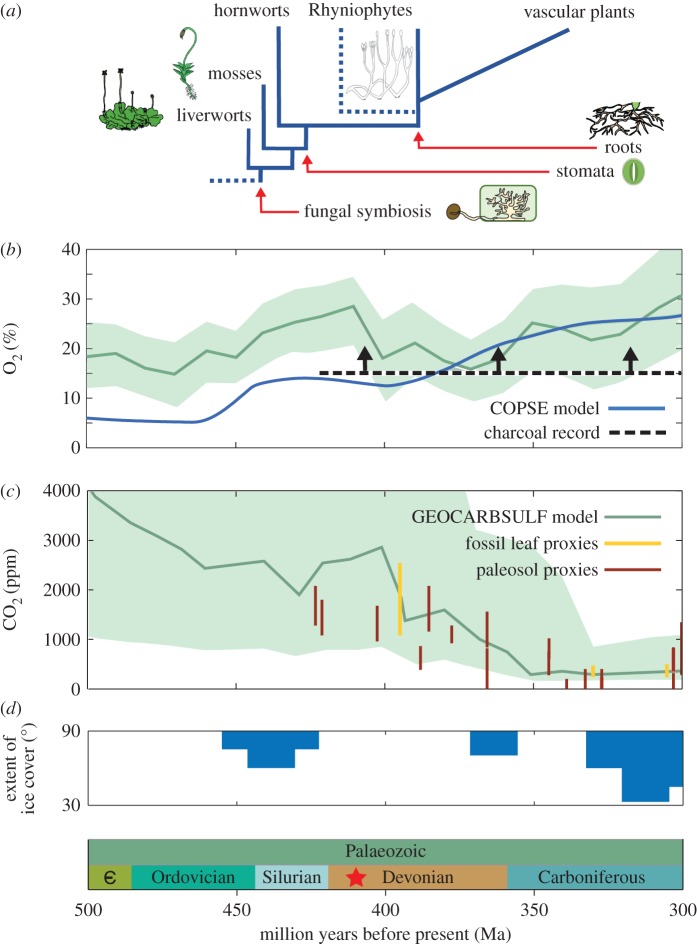
Plant evolution and climate during the Palaeozoic Era. (*a*) The divergence of early non-vascular plants and timings of the development of fungal symbiosis, stomata and roots. (*b*) Model predictions for atmospheric oxygen concentration (green line and shading for GEOCARBSULF model [1]; blue line for COPSE model [2,3]) alongside the lower limit defined by the presence of fossil charcoal (black dashed line [4]). (*c*) The reconstructions of atmospheric CO_2_ concentration from modelling and proxies (colours the same as (*b*); maroon lines for palaeosol proxies [5]; yellow lines for fossil leaf proxies [6], see text). (*d*) The extent of ice cover shown as palaeolatitiude of ice caps [7].

The first plants to colonize the Earth's land surface (during the Palaeozoic Era, 541–250 Ma) faced an entirely different climate to today (figure 1), with atmospheric CO_2_ concentrations being greater than 1000 ppm [5]. While modern plants flourish under elevated CO_2_, access to mineral nutrients likely posed a problem for the early terrestrial biosphere—the earliest land-colonizing plant species lacked roots, being non-vascular and liverwort-like [8–11] (figure 1*a*), and the substrate onto which they emerged was a skeletal mineral soil, largely lacking in organic matter [12]. Fossil beds at the Rhynie chert provide evidence that these early plants formed symbioses with fungi [13–15], which are likely to have facilitated mineral nutrient acquisition, in particular phosphorus [16,17]. It is likely that the nutrient acquisition strategies via fungal symbiosis had a significant effect on global primary productivity, and therefore climate, but these aspects have not been explored in detail.

**Figure 2.**
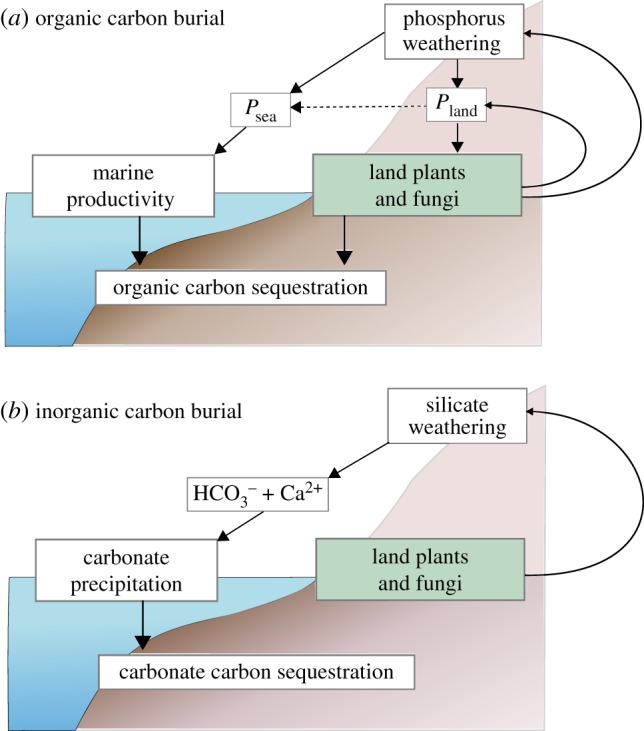
Model diagram for the effects of land plant–fungal symbioses on weathering and the consequences for organic (*a*) and inorganic carbon burial (*b*). (*a*) Continental weathering supplies phosphorus that is either taken up on land or transported to the ocean. Both routes enhance primary productivity and result in the eventual burial of organic carbon, which decreases atmospheric CO_2_ and produces O_2_. Plants and fungi can modify both the overall phosphorus weathering flux and the uptake of phosphorus on land. The dashed line indicates that increasing *P*_land_ acts to reduce *P*_sea_. (*b*) Biotic effects on the weathering of silicate minerals result in further sequestration of carbon as sedimentary carbonates (see text).

Plants play a key role in the biogeochemical cycles of carbon, phosphorus and oxygen, and this role is amplified by symbiosis with fungi (figure 2). In exchange for photosynthetically fixed carbon, mycorrhizal fungi provide plants with mineral nutrients that would be otherwise inaccessible [17]. Plant tissue contributes a net carbon sink when organic matter is buried in sediments, and burial of this reduced organic carbon results in a net source of O_2_ to the atmosphere, which has been the major mechanism of oxygen production over Earth history [18]. Plants and mycorrhizal fungi also drive additional drawdown of atmospheric CO_2_ through their enhancement of silicate weathering [19], which occurs through acidification of the soil environment [20] and exudation of organic acids directly from plant roots and indirectly from fungi. Silicate weathering transfers atmospheric CO_2_ into solution as bicarbonate, alongside cations (e.g. Ca^2+^), which eventually combine in the oceans to form sedimentary carbonates (figure 2).

Biotic weathering also liberates inorganic phosphorus from rocks and, in turn, stimulates plant growth and microbial activity [21–23]. Terrestrially derived phosphorus forms the riverine input to the ocean, which further promotes the global burial of organic carbon through the enhancement of marine photosynthetic primary productivity (e.g. by phytoplankton). Only a small fraction of the global phosphorus supply is ultimately buried within terrestrially derived organic matter; the majority of phosphorus exits the system with marine organic carbon or as authigenic sedimentary minerals [24], but because plants produce structural components such as lignin that are lower in phosphorus than marine organic matter, terrestrial phosphorus acquisition results in greater carbon burial in terrestrial systems relative to marine systems for the same amount of phosphorus [25].

Multiple proxies indicate that the global environment underwent major changes during the Palaeozoic phase of plant colonization, which supports a link between terrestrialization of plants and biogeochemical cycling (figure 1). Global biogeochemical models that link plants, carbon cycling and climate, although uncertain, generally predict a decline in CO_2_ concentrations over the Palaeozoic Era, coincident with the development of the terrestrial biosphere [1–3,19]. This CO_2_ drawdown is driven by the modelled biotic enhancement of silicate mineral weathering and an increased burial rate of organic carbon (both terrestrial and marine) due to greater phosphorus availability. Proxies that attempt to infer atmospheric CO_2_ concentrations based on palaeosols [5,26] and fossil leaves [6] also carry considerable uncertainty, but agree with models in showing a decline in CO_2_ concentrations during the Palaeozoic and reaching roughly modern levels in the Carboniferous period. These approaches indicate that low atmospheric CO_2_ concentrations, in combination with the reduced incoming radiation from the fainter sun, resulted in the approximately 300 Ma Permo-Carboniferous ‘icehouse’, a deep glacial period in which ice sheets extended into the subtropics (figure 1*d*; [7]). The driving factors underpinning earlier glacial periods are far less well understood, particularly given the uncertainties in atmospheric CO_2_ concentrations. Several hypotheses have been proposed to explain the Late Ordovician Hirnantian glaciation, including a reduction in volcanic CO_2_ input [27] and/or a tectonically driven enhancement to silicate weathering rates [28], but this glaciation has also been attributed to the evolution and biogeochemical effects of the early terrestrial biosphere [22].

The Rhynie chert, which formed during the Lower Devonian (411 ± 1.3 Ma; [29]), documents a period of considerable uncertainty in the Earth's history in terms of climate and biosphere response and has been central to our understanding of the evolution of plant–fungal symbioses. The unique geological features present in the environment at the time of formation [30] produced a fossil bed containing uniquely well-preserved plant specimens that contain fossilized fungal structures within the plant tissue [13,31,32]. These fungal structures bear strong structural homology to the mycorrhizal and mycorrhiza-like associations formed between modern-day land plants and Glomeromycotina fungi [13]. This type of fungal associate is found in more than 85% of land plants today [33], spanning the land plant phylogeny (figure 1*a*). The discovery of mycorrhiza-like fungi in Rhynie chert fossils provided support for the long-standing hypothesis that symbiotic fungi played a vital role in helping plants colonize the terrestrial environment more than 475 Ma [34].

Recent evidence indicates that the Glomeromycotina were not the only fungus to form symbioses with plants at the first stages of land plant evolution. The earliest diverging liverworts, the Haplomitriopsida, form associations with fungi of a clade that is thought to have diverged earlier than the Glomeromycotina—the Mucoromycotina [35]. Since this discovery, experiments have revealed that, like the Glomeromycotina, the liverwort–Mucoromycotina associations are nutritionally mutualistic [36] and that the associations are present in taxa throughout the land plant phylogeny [37–40]. Re-examination of Rhynie chert fossils has revealed that early Rhyniophytes (*Horneophyton*, *Nothia*) were frequently colonized by at least two fungal endophytes bearing morphological characteristics similar to those of modern Glomeromycotina and Mucoromycotina fungal associations in extant plants, indicating that both fungal symbionts may have been present during initial plant terrestrialization [41,42]. It has since been shown that such dual, nutritionally mutualistic colonizations are also common throughout the plant kingdom [37,43].

**Figure 3.**
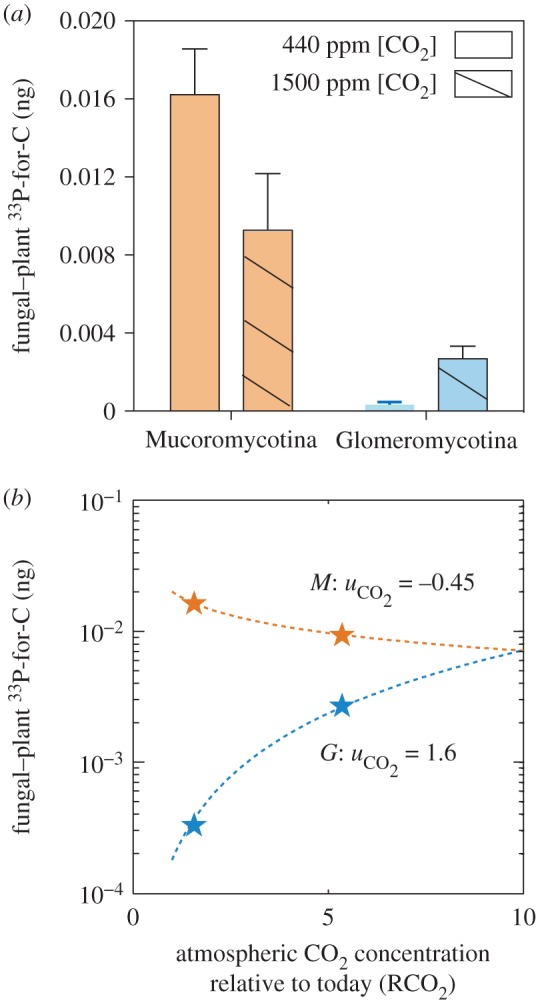
Experimental observations of plant–fungal phosphorus and carbon exchange in liverworts with Mucoromycotina and Glomeromycotina fungal partners [36,44]. Experiments were carried out under ambient and simulated Palaeozoic, elevated CO_2_ concentrations. (*a*) Phosphorus-for-carbon exchange (nanograms) at ambient (440 ppm, open boxes) and elevated (1500 ppm, hashed boxes) CO_2_ concentrations. (*b*) Functional fits of fungal plant phosphorus-for-carbon exchange data. Data points are shown as stars, functional fits are shown as the dotted line for Mucoromycotina (orange) and Glomeromycotina (blue) (see text). 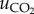 is a parameter representing the strength of the relationship between atmospheric CO_2_ and P-for-C exchange (see text).

Recent experimental evidence has shown that the mutualistic functioning of each type of mycorrhiza-like association is affected differently by a high-CO_2_ atmosphere in terms of carbon-for-phosphorus exchange between symbionts. Liverworts associated only with Glomeromycotina fungi acquire greater amounts of phosphorus via their fungal partner under a simulated high Palaeozoic CO_2_ concentration (1500 ppm) compared to modern, relatively low, ambient CO_2_ concentrations (440 ppm) [44]. In contrast, liverworts that form partnerships only with Mucoromycotina fungi, or with both fungal types simultaneously, show the opposite response (figure 3; [36,43]). When coupled with palaeontological and molecular evidence, these findings lead to the hypothesis that Mucoromycotina–liverwort associations could be ancestral, or are at least as ancestral as liverwort–Glomeromycotina symbioses, and that early land plant fungal associations were more transient and varied than previously assumed [44]. However, the impact and feedbacks on the Palaeozoic global climate of the presence of mutualistic CO_2_-responsive mycorrhizal symbionts remain unexplored.

Given the significance of the role of symbiotic soil fungi in modern terrestrial phosphorus cycling, it is likely that the evolution of plant–fungal symbioses was critical for acquisition of nutrients by the burgeoning land flora. This means that plant–fungal symbioses may therefore have helped to drive a shift from marine to terrestrial productivity and a corresponding increase in the global rate of organic carbon burial. However, the degree to which early plants in symbiosis with mycorrhizal fungi were able to accelerate silicate and phosphorus weathering is uncertain, and, while experiments with liverworts and bryophytes have shown significant enhancements to weathering, it is difficult to extrapolate these effects to the global phosphorus supply [22,23], although methods are improving [45]. We hypothesize that changes to plant–fungal phosphorus acquisition will have distinct effects on terrestrial net primary productivity (NPP), CO_2_ drawdown and oxygen production and that these effects will vary according to fungal symbiont identity/function.

In this paper, we test this hypothesis by incorporating existing physiological data from experiments using early diverging liverwort species and their native fungal symbionts (figure 3) into a global biogeochemical model, which approximates the biogeochemical processes shown in figure 2. This approach allows us to better understand the potential impact of early plant–fungal phosphorus acquisition on global climate and biogeochemical cycling during the Palaeozoic Era.

### (a) Insights from plant–fungal physiology

The unexpected diversity in fungal symbiont identity and functioning across land plants has implications not just for the evolution of the terrestrial biosphere, but also for feedbacks on the Earth's climate. In Field *et al*. [36,44] carbon-for-phosphorus exchange between liverworts and their fungal symbionts was measured at both modern ambient (440 ppm [CO_2_]) and simulated Palaeozoic (1500 ppm [CO_2_]) atmospheres under controlled environment conditions. To determine the movement of phosphorus from fungus to plant, ^33^P-orthophosphate solution was supplied to fungus-only compartments in pot-based microcosms and incubated for 21 days. After harvest, ^33^P activity was measured in plant tissues using acid digestion and liquid scintillation. In the same experimental microcosms, ^14^CO_2_ was supplied to the liverworts for one complete photoperiod before being measured in the fungus-only compartments within the pots through sample oxidation and liquid scintillation (full methodological details are published in [36,44]). Total carbon and ^33^P budgets for each microcosm were calculated using published equations (from Cameron *et al*. [43,46], respectively).

The results from these experiments show that liverworts with Glomeromycotina fungal partners gain greater amounts of ^33^P per unit of plant-fixed carbon transferred to the fungus under a Palaeozoic atmospheric CO_2_ concentration than when compared with a modern ambient CO_2_ atmosphere, but liverworts with Mucoromycotina fungal symbionts display the opposite trend (figure 3*a*; [36,43]).

## 2. Material and methods

We examined the implications of contrasting plant–fungal relationships (figure 3) in response to changes in CO_2_ by incorporating phosphorus effects on global plant biomass and the ability of fungi to affect plant phosphorus uptake into the COPSE biogeochemical model (Carbon Oxygen Phosphorus Sulphur Evolution) [2,3].

The COPSE model [2,3] reconstructs the long-term cycling of carbon, oxygen, phosphorus and sulfur between a simplified representation of the land biota, atmosphere, oceans and sediments. A key component of the model is the terrestrial vegetation, which is assumed to take up a fraction of total weathered phosphorus. The vegetation produces organic carbon (that is eventually buried) and enhances terrestrial weathering rates (figure 2). Global terrestrial biomass is represented in COPSE by a single variable *M*_bio_, which comprises constraints based on the expansion of the terrestrial biosphere onto the land surface (*E*), limitation by surface temperature (*V*_T_), prevalence of wildfires (*V*_fire_) and the atmospheric concentrations of CO_2_ (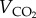) and O_2_ (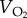 ), which affect CO_2_-fertilization and photorespiration. We adapt the COPSE model to allow for the terrestrial phosphorus supply (*V*_P_) to explicitly affect the mass of the biosphere, in order to represent the effects of phosphorus limitation on both carbon burial and chemical weathering:
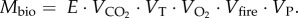
The phosphorus supply to the land biota (*V*_P_) is calculated from the available weathered phosphorus (*phosw*) multiplied by an uptake efficiency parameter (*k*_uptake_), which allows for possible fungal-driven changes to plant phosphorus acquisition:
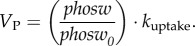
Here, the constant *phosw_0_* is the present-day rate of phosphorus weathering used to normalize the expression. The climatic limiting factors are defined to constrain biomass in the following ways, and are unaltered from the original COPSE model [2]:
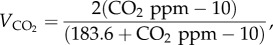

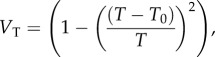

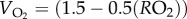

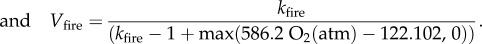
All other equations are the same as in the original model, except for the expression for phosphorus buried with land plant matter (*P*_land_), which is amended to ensure that it cannot exceed phosphorus supply from weathering:
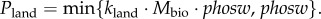
Here, *k*_land_ is the present-day fraction of weathered P buried on land. Land-derived organic carbon burial (locb) is calculated from the relative value of *P*_land_ and a present-day biomass burial rate (*k*_locb_) (assuming a present-day C : P ratio for plant material):
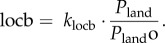
All weathered phosphorus that is not used on land is transferred to the ocean (*P*_sea_), where it stimulates marine productivity.
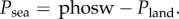


Our modified Fungal COPSE (Fun-COPSE) model allows us to observe the possible long-term climate effects associated with changes in plant phosphorus uptake efficiency, and how this may influence our reconstructions of Paleozoic climate. In particular, our introduction of a direct phosphorus limitation of the terrestrial vegetation introduces a positive feedback loop to the model (figure 2*a*) wherein terrestrial productivity increase phosphorus weathering and uptake, which in turn further increases productivity.

We experiment by running the model with *k*_uptake_ defined following the relationships between CO_2_ concentration and phosphorus-for-carbon exchange efficiency observed in experiments with Mucoromycotina and Glomeromycotina fungi. Figure 3*c* shows representative curves that are fitted to the experimental data [43], which take the form 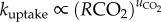, where *R*CO_2_ is the relative atmospheric CO_2_ concentration. For Glomeromycotina, we require 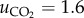 and for Mucoromycotina, we require 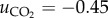.

More experimental data are needed to further constrain the relationships between fungal identity, phosphorus acquisition and climate, but the present formulation allows us to assess the potential climate impacts of a variable terrestrial phosphorus uptake efficiency (*k*_uptake_). The assumed relationship for Glomeromycotina is particularly strong, based on the observed order-of-magnitude increase in phosphorus-for-carbon exchange at the elevated CO_2_ concentration. The strength of the relationship may be overestimated in the experimental systems. In nature, the fungus forms symbioses and provides phosphorus to multiple plant partners. In addition, the fungus likely competes for phosphorus resources with diverse soil microbial communities. As a result of this, and together with the observation that such a high phosphorus uptake efficiency results in numerical instability in the model due to positive feedbacks, we reduce the maximum value of 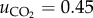, which mirrors the magnitude of the Mucoromycotina relationship. By exploring the range of phosphorus-for-carbon use efficiencies, we capture the uncertainty in scaling local plant–fungal–soil interactions to the global level and at geological timescales.

The most recent version of COPSE [3], which we have modified here, experiments with a plant-driven enhancement to the efficiency of phosphorus weathering (i.e. more phosphorus weathered per bulk rock weathering) and an assumed increase in the carbon buried per unit phosphorus for early plants, based on high C : P ratios measured in bryophytes. These modifications are admittedly speculative, and form the maximum error window in the aforementioned model results. For the current work, we use the more conservative version of the model (shown in green in Lenton *et al*. [3] and plotted in figure 1) in which global phosphorus weathering depends only on bulk rock weathering rates.

We use our model to examine how the nature of plant phosphorus uptake via fungal partners (i.e. our parameter *k*_uptake_) influences modelled NPP, atmospheric CO_2_ and O_2_ concentrations, and global temperature in response to land colonization during the Palaeozoic Era.

## 3. Results and discussion

**Figure 4.**
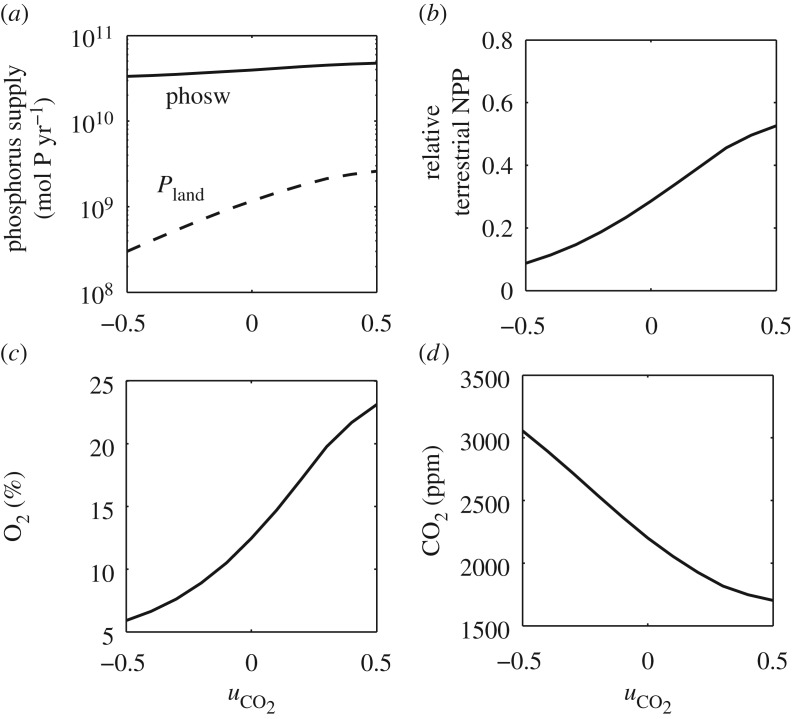
The effect of phosphorus uptake efficiency 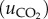 on global environmental conditions. Panels represent modified COPSE model steady states at 410 Ma for a range of phosphorus uptake efficiency parameters fixed at each value for 410 Ma. The efficiency of phosphorus uptake is modelled as 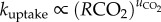 (see text). The panels represent phosphorus uptake efficiency's effect on (*a*) total phosphorus weathering input (phosw) and terrestrial uptake (*P*_land_), (*b*) terrestrial NPP relative to today, (*c*) atmospheric O_2_ and (*d*) atmospheric CO_2_.

Broadly, Fun-COPSE shows that changes to plant–fungal phosphorus uptake, such as those observed in laboratory conditions under high atmospheric CO_2_, could have a large effect on the modelled Palaeozoic climate transition. Model steady states at 410 Ma indicate that altering the efficiency of terrestrial phosphorus uptake changes the amount of phosphorus taken up on land, without requiring a significant change to the overall phosphorus weathering rate (figure 4*a*). Relative terrestrial NPP scales with the supply of the limiting nutrient phosphate, and therefore is also significantly different between steady states (figure 4*b*). Efficient phosphorus uptake at superambient CO_2_ results in enhanced organic carbon sequestration, which contributes to a reduction in CO_2_ (figure 4*c*) and drives a rise in atmospheric O_2_ (figure 4*d*). Assuming less efficient phosphorus uptake at high CO_2_, as observed in liverwort–Mucoromycotina symbioses [36], results in model predictions of higher CO_2_ and lower O_2_ concentrations in the atmosphere (figure 4*c*,*d*).

**Figure 5.**
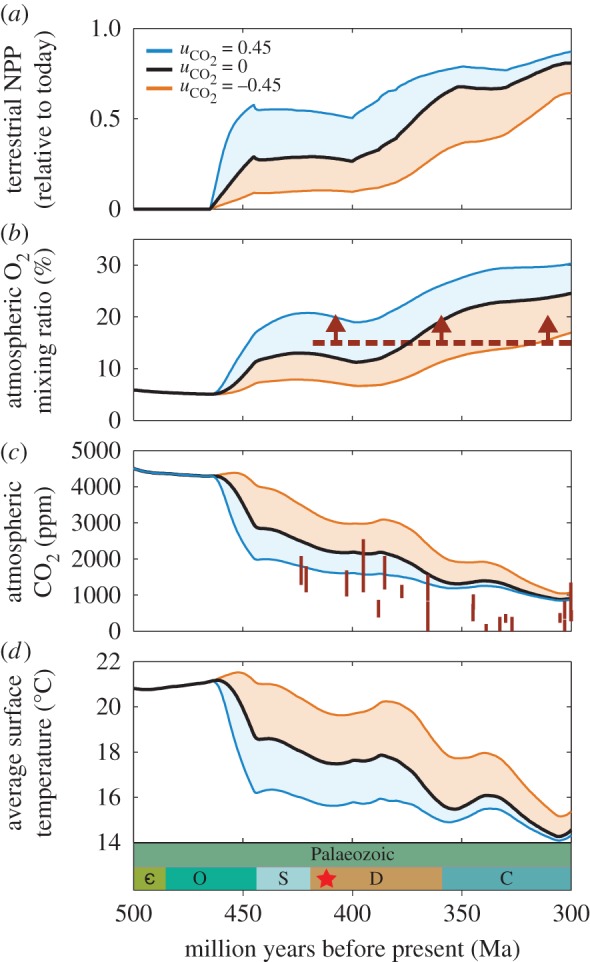
The effect of plant–fungal-derived phosphorus on the global environment throughout the Palaeozoic Era. Terrestrial phosphorus uptake is modelled based on the observed carbon-for-phosphorus exchange ratios for either Glomeromycotina (blue) or Mucoromycotina (orange). The shaded area of the red or blue lines indicate the effect of such symbioses with a range of phosphorus uptake efficiencies. The effect of phosphorus uptake efficiency on (*a*) the relative terrestrial NPP, (*b*) atmospheric O_2_, with charcoal record as in figure 1, (*c*) atmospheric CO_2_, with proxies as figure 1 and (*d*) global average surface temperature. Present-day average surface temperature is around 15°C, which we assume is typical of an ‘icehouse’ climate (i.e. the presence of ice caps, such as in the modern world, and during the Late Ordovician glaciation).

The time-integrated model predictions for the Early Palaeozoic are similarly affected by phosphorus uptake efficiency (figure 5). As in the previous version of the COPSE model, Fun-COPSE broadly recreates the global environmental changes thought to have occurred during the Palaeozoic Era. The expansion of the early terrestrial biosphere leads to enhancements of terrestrial weathering, which buries more carbon via the silicate–carbonate cycle and by increased delivery of phosphorus to both terrestrial and marine systems. When we include changes to the fungal phosphorus uptake however, the model predicts large differences in the timing and degree of these environmental changes (orange and blue lines in figure 5). The range of uncertainty in all reconstructed parameters is high, confirming the power of the phosphorus cycle to alter model predictions in COPSE [3].

Further uncertainty remains regarding the productivity of the early terrestrial biosphere, and the effects of early plants on the global carbon cycle and climate. This topic has been the focus of considerable debate in the literature, which has recently turned to the question of the ability of early terrestrial plants to enhance silicate weathering rates and liberate phosphorus [3,22,23]. Our findings indicate that, in addition to these considerations, the nature of the supply of phosphorus to plants from fungal partners can contribute significantly to the wider environmental changes that occurred during the Palaeozoic (figure 5).

Assuming a phosphorus uptake efficiency greater than that observed today, as might be expected if Glomeromycotina were the dominant fungal symbionts of early land plants (figure 5, blue lines), leads to a highly productive early land biosphere. This is capable of drawing down CO_2_ to such a degree that global average surface temperature may be sufficiently low to trigger the Late Ordovician glaciation, without requiring additional tectonic considerations. In this case, atmospheric oxygen concentration rises to modern levels by approximately 420 Ma, coincident with the first evidence for wildfires [47]. However, assuming a phosphorus uptake efficiency lower than present day, as might be the case if Mucoromycotina fungal associates were the predominant symbiont within the land flora (orange lines), limits productivity. In this scenario, CO_2_ concentration and temperature remains high throughout the early colonization phase, and O_2_ remains less than 10% atm.

These scenarios show entirely different drivers for Palaeozoic environmental change, with the latter implying that the early terrestrial biosphere may not have been responsible for either glaciation or substantial O_2_ rise. However, the low-productivity scenario is in conflict with available evidence (figure 5*b,c*), thus if plant phosphorus uptake was suppressed globally under high CO_2_, then one must appeal either to an increased efficiency of phosphorus weathering or to currently unknown tectonic or palaeogeographic factors. Nevertheless, such extremes in model output show that the ability of the terrestrial biosphere to acquire phosphorus, rather than simply its ability to affect continental weathering, must be taken into account in future investigations.

It is likely that the earliest land plants were cosmopolitan in their choice of fungal symbionts [44], because different fungi may have provided multiple, additional and non-nutritional, benefits. These benefits are likely to have included enhanced access to water and resistance to disease and/or herbivores, as is the case with their extant relatives [48,49]. It seems probable that plants would have associated with a variety of—and potentially a multitude of—partners at different stages of their evolution and spread. Therefore, the relative dominance of Glomeromycotina or Mucoromycotina fungi within early plant assemblages may have been transient according to climate and/or ecology. By varying the strength of phosphorus-for-carbon efficiency 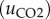, Fun-COPSE allows for two possible symbiotic scenarios: (i) multiple simultaneous symbiotic partners within individual plants, and/or (ii) the co-existence of multiple plants with different symbiotic partners across the global land surface. Thus, the overall function of the land vegetation is unlikely to reflect the operation of any single plant–fungal system explored here.

A greater understanding of the magnitude and balance of fungal partnering requires further experiments conducted on extant examples of early diverging plants with a suite of fungal symbionts at a range of atmospheric CO_2_ concentrations reflective of conditions at various points in the Earth's history. These experiments should be designed to allow quantification of the relative costs and benefits to plants and fungi of symbiosis, with the results being used to construct models of the terrestrial biosphere that include phosphorus-for-carbon exchange and phosphorus recycling.

Our findings show significant Earth system sensitivity to phosphorus uptake from mycorrhizal fungi. Current dynamic global vegetation models (e.g. SDGVM [50]) linked to detailed spatial palaeoclimate reconstructions have been shown to respond in different ways to simple models of the long-term carbon cycle (e.g. COPSE, GEOCARB [51]). Spatial models of non-vascular vegetation have elucidated the potential for high productivity in the early terrestrial biosphere [45], yet current vegetation modelling efforts are hampered by the inclusion of only rudimentary phosphorus cycling and associated mycorrhizal functioning [52–54], which as we show here, may be of great importance.

Fossil evidence from the Rhynie chert has provided unique insights into the plant–fungal relationships during the early period of plant evolution and has allowed for the focused experimental study of how these partnerships may have functioned in response to changing atmospheric CO_2_ concentrations. Uncovering the nature of the Palaeozoic climate transition, and therefore the emergence of the human-habitable world, relies heavily on understanding the ability of the early terrestrial biosphere to acquire and use phosphorus. Such advances must come from a linked campaign of experiments and trait-based modelling, informed by palaeobotanical studies on preserved ancient ecosystems, such as the Rhynie chert. Efforts must also be made to integrate aspects of the geochemical literature, such as the relationships between erosion, soil shielding and global chemical weathering fluxes [55].

## 4. Conclusion

The global terrestrial biosphere response to high CO_2_ depends greatly on the functioning of plant–fungal symbioses and phosphorus cycling. The experiments of Field *et al*. [36,43] show that these responses are not straightforward. Efforts to understand the plant-driven Palaeozoic climate transition using models would benefit from the inclusion of a mechanistic representation of plant–fungal phosphorus and carbon exchange that is formulated from empirical data, and the resulting predictions should be independently tested against geochemical data. Our initial effort to incorporate fungal phosphorus uptake into an existing biogeochemical model is based on limited experimental results and extrapolation to large spatial and temporal scales, which is necessarily a first step that must be improved upon. Nevertheless, we show that the effect on the modelled climate transition may be highly significant, and reliant upon the nutritional nature of the relationship between plants and symbiotic fungi. Our findings raise questions about current reconstructions of plant-driven changes to climate during the Palaeozoic, and show that understanding the mechanisms of global plant phosphorus uptake, rather than just the liberation of phosphorus during continental weathering, is essential for resolving past climatic and environmental changes throughout the Earth's history.

## Data accessibility

This article has no additional data.
